# Spherical Coordinate System for Dyslipoproteinemia Phenotyping and Risk Prediction

**DOI:** 10.3390/jcm14217557

**Published:** 2025-10-24

**Authors:** Justine Cole, Maureen Sampson, Alan T. Remaley

**Affiliations:** 1 Lipoprotein Metabolism Laboratory, Translational Vascular Medicine Branch, National Heart, Lung, and Blood Institute, National Institutes of Health, Bethesda, MD 20892, USA; 2 Clinical Center, Department of Laboratory Medicine, National Institutes of Health, Bethesda, MD 20892, USA

**Keywords:** dyslipoproteinemia, dyslipidemia, cardiovascular risk assessment, spherical coordinates, logistic regression

## Abstract

**Background/Objectives**: The factors contributing to residual atherosclerotic cardiovascular disease (ASCVD) risk in individuals are not fully understood, but knowledge of the specific type of dyslipoproteinemia may help further refine risk assessment. We developed a novel phenotyping and risk assessment system that may be applied automatically using standard lipid panel parameters. **Methods**: NHANES data collected from 37,056 individuals during 1999–2018 were used to develop a three-dimensional dyslipidemia phenotype classification system. ARIC data from 14,632 individuals were used to train and validate the risk model. Three-dimensional Cartesian coordinates were converted to spherical coordinates, which were used as features in a logistic regression model that provides a probability of ASCVD. UK Biobank data from 354,344 individuals were used to further validate and test the model. **Results**: Nine lipidemia phenotypes were defined based on the concentrations of HDLC, non-HDLC and TG. These phenotypes were related to the prevalence of metabolic syndrome, pooled cohort equation (PCE) score and ASCVD-free survival. A logistic regression model including age, sex and the spherical coordinates of the phenotype provided a composite risk score with predictive accuracy comparable to that of the PCEs. **Conclusions**: We provided an example of how a multidimensional coordinate system may be used to define a novel lipoprotein phenotyping system to examine disease associations. When applied to an ASCVD risk model, the composite spherical coordinate risk marker, which can be fully automated, provided an F1 performance score almost as good as the PCEs, which requires other risk factors besides lipids.

## 1. Introduction

Atherosclerotic cardiovascular disease (ASCVD) is a complex, chronic disorder involving several pathogenic processes that all interact with each other. A key convergence point, and final common pathway, of atherosclerosis is the entrapment of apoB-containing lipoproteins in the arterial intima, where it can accumulate either inside cells or in the extracellular space as cholesterol crystals, which together can cause biomechanical and cellular damage, resulting in inflammation and ongoing dysfunctional responses. Because most circulating cholesterol is carried on low-density lipoproteins (LDL), LDL-cholesterol (LDLC) is commonly used as the primary marker of cardiovascular disease (CVD) risk and to triage patients for lipid-lowering treatment. Nevertheless, recent evidence has revealed that atherosclerosis is often still present in patients with “normal” or even optimally lowered concentrations of LDLC [1,2].

To improve CVD risk assessment, several risk factors, including total cholesterol (TC) and high-density lipoprotein cholesterol (HDLC) and other risk factors like hypertension, diabetes and smoking status are combined into risk scores, such as the pooled cohort equations (PCEs) that are recommended in the US guideline on the management of blood cholesterol [3]. In addition, other risk-enhancing factors, such as inflammatory markers and conditions, triglyceride (TG) and lipoprotein (a) (Lp(a)) can also be considered in making decisions on statin therapy for patients with intermediate risk by the PCE risk score [3]. It has been shown, however, that population-based risk scores often fall short of accurately predicting the development of atherosclerosis and coronary events [2,4,5]. Furthermore, the current CVD risk scores are typically not automatically calculated and thus likely have variable compliance in routine clinical practice.

Dyslipoproteinemia phenotyping is a potential way to examine in more detail how genetic predispositions and environmental risk factors interact to result in cardiovascular disease. Fredrickson, Levy and Lees (FLL) first described six dyslipoproteinemia phenotypes based on abnormalities in any combination of chylomicrons (CM), very low-density lipoproteins (VLDL), remnant lipoproteins and low-density lipoproteins (LDL) [6]. Such lipoprotein phenotyping can expand the clinician’s appreciation of the underlying pathology and may alter prognosis and affect clinical management. Unfortunately, patients are rarely categorized or diagnosed in this manner, owing to the unavailability of the requisite testing methods, such as lipoprotein electrophoresis. De Graaf et al. proposed a useful algorithm to determine similar phenotypes based on apolipoprotein (apo)B, TC and TG measurements [7], and Gilliland et al. used this algorithm to confirm that coronary artery disease risk varies with the phenotype [8].

To assist clinicians in the interpretation of lipid CVD risk biomarkers, some clinical laboratories report ratios, such as TG/HDLC, TC/HDLC and LDLC/HDLC, which provide information on the pattern of dyslipoproteinemia, even in the absence of dyslipidemia [9,10]. Reporting of the ratios proposed by De Graaf et al. (TG/apoB and TC/apoB) would allow routine use of their lipoprotein phenotyping method but relies on measurement of apoB, which is still not routine practice, despite the demonstrated value of apoB over LDLC [11]. In addition, health care providers, especially in the primary care setting, are unlikely to perform detailed CVD risk calculations or to follow some of the more complex algorithms that are recommended by some CVD guidelines [3,12,13].

We recently reported on two relatively simple CVD risk-scoring algorithms, using the standard lipid panel and clinical data that is routinely available to the clinical laboratory, which could be automatically reported on all patients for CVD risk management. The first is an equation to produce an estimated risk score that approximates the PCEs [14]. The second is a two-dimensional phenotyping method, using non-HDLC and TG to predict the Fredrickson—Levy—Lees (FLL) class of dyslipoproteinemia, except Type III, which requires apoB measurement [15]. Given the importance of HDLC as a negative risk marker, we aimed here to develop a new automated phenotyping system that utilizes all three of the main lipid parameters (non-HDLC, TG and HDLC) estimated in a standard lipid panel. We also used these parameters to derive spherical coordinates, which allow for intuitive visualization of the dyslipidemia as an expanding sphere, with the radius, r, indicating severity. Spherical coordinates also account for the potential non-linear interactions among these parameters and their impact on CVD risk. The spherical coordinates were thus used to develop a composite risk score from the standard lipid panel and available clinical data. Our aim in this was to exemplify the application of a multidimensional spherical coordinate system that could potentially reflect the non-linear impacts of varying lipid parameter values in CVD risk assessment. In addition, we wanted to use this new risk score, along with age and sex, which are routinely available in clinical laboratories, to develop a risk score that could be automatically reported by the clinical laboratory on all patients tested with a lipid panel.

## 2. Materials and Methods

### 2.1. Study Design and Data Acquisition

Deidentified clinical laboratory data, demographic and clinical data were extracted from the National Health and Nutrition Examination Survey from years 1999–2018 (NHANES; n = 37,056) [16], from the Atherosclerosis Risk in Communities dataset (ARIC; n = 14,632) [17], and from UK Biobank (n = 354,344) [18]. Atherosclerotic cardiovascular disease outcome information was also extracted from ARIC and UK Biobank. Only those between 40 and 70 years were included, and those on lipid-lowering medications were excluded. The NHANES data were used to develop a novel dyslipidemia phenotyping system. As ASCVD outcome data is not available in NHANES and outcomes are poorly balanced in UK Biobank, the ARIC data were used to train and validate logistic regression models that provide a probability of ASCVD. The UK Biobank data were used to test the models. For time-to-event analysis, patients in ARIC were followed for up to 32 years, and in UK Biobank, for a mean of 10 years and maximum of 15.5 years. LDLC was calculated using the Sampson Equation [19]. Only deidentified data from publicly available databases were used for analysis and hence the current study was considered non-human subject research.

### 2.2. Definition of ASCVD and Metabolic Syndrome

The metabolic syndrome (MetS) was defined using the 2004 American Heart Association definition [20], and ASCVD was defined as all cardiovascular disease except heart failure. In the ARIC dataset, ARIC physicians adjudicated events in hospital records and assigned a standardized classification. In UK Biobank, ICD10 codes were used to classify ASCVD (Appendix A).

### 2.3. Phenotype Classification

**Figure 1:**
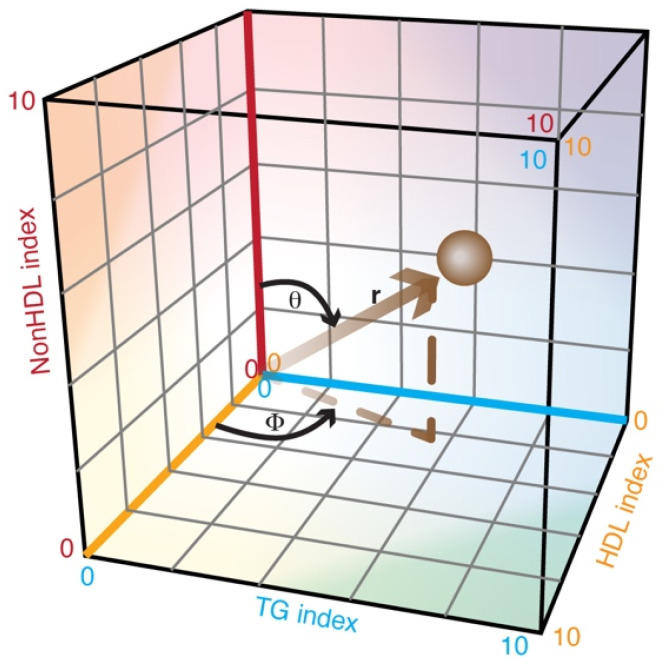
Conversion of lipid indices to spherical coordinates, where r is the length of the vector from the origin to the datapoint, and θ and φ are the polar and azimuthal angles, respectively.

**Figure 2:**
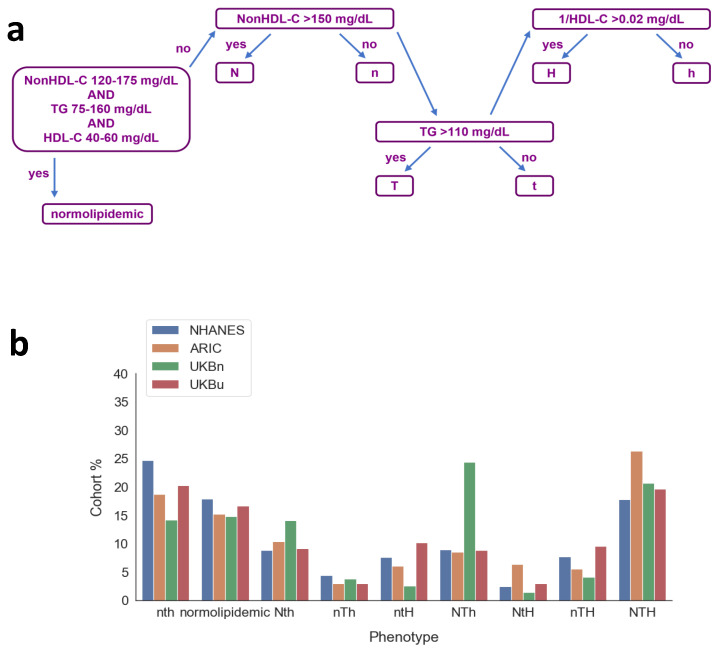
(**a**) Algorithm for determining a novel 3-letter phenotype with upper-case letters denoting values above the median and lower-case letters denoting values below the median, or normolipidemic if within the interquartile range of the NHANES data. (**b**) Distribution of data from each database among the 9 novel phenotypes.

The NHANES dataset was used to create a novel phenotyping classification system, based on NHDLC, TG and HDLC (Figure 1 and Figure 2a). The NTH phenotyping system classifies individuals as normolipidemic if their NHDLC is 120–175 mg/dL, their TG is 75–160 mg/dL and their HDLC is 40–60 mg/dL, based on the NHANES interquartile ranges (IQRs) for each parameter (Table 1). If not normolipidemic, then the individual receives either a capital or a lower-case letter for each of NHDLC, TG and 1/H, depending on whether their result is above (capital) or below (lower-case) the rounded median for each, i.e., NHDLC: 150 mg/dL; TG: 110 mg/dL; 1/H: 0.02 dL/mg. Nine phenotypes were thus described. This system was applied to the NHANES, ARIC and UK Biobank datasets. The groups were also recalculated in UK Biobank using the UK Biobank medians and IQRs. We analyzed the prevalence of MetS, the PCE scores and ASCVD-free survival by phenotype in each dataset.

We wished to visualize the data in three-dimensional space to provide further nuance that is not captured in the labeling system. TG data were log-transformed (lnTG) to provide a normal distribution and the inverse of HDLC was taken (1/H) to ensure positive correlations between each pair of lipid features. The means and standard deviations of each feature were determined and were used to transform the values of each feature into z-scores. The data was truncated to include only those instances with all values in the range −5–5. The data was then shifted into the first (+,+,+) octant in three-dimensional space by adding 5 to all values.

### 2.4. Development of Spherical Coordinate Metrics and Model

With only positive values for all Cartesian coordinates of 1/H (x), lnTG (y) and NHDLC (z), the spherical coordinates were calculated as follows:

r = (x2 + y2 + z2) 0.5

Theta (θ) = arrcos(z/r)

Phi (φ) = arctan(y/x)

where r is the length of the vector from the origin to the datapoint, and θ and φ are the polar and azimuthal angles, respectively (Figure 1).

The ARIC dataset was used to create three logistic regression models that predict ASCVD. Model 1 included only the spherical coordinates as features. Model 2 included L1, the probability output of Model 1, and sex, and produced output, L2. Model 3 was the same as for Model 2 but included age as a third feature and gave the output index, L3. For all three models, the final probability output (0–1) was multiplied by 100 to yield the final lipid index score. The models were adjusted, using the logit shift method, before application to UK Biobank data, in which the prevalence of ASCVD is low. An adjustment was not made for NHANES in which the prevalence is unknown.

### 2.5. Statistical Analysis

ASCVD-free survival was examined by phenotype and by quintiles of LDLC, PCE score and L3. The area under the receiver operating characteristic (AUROC) curve for ASCVD prediction was used to compare risk markers, including the outputs of our three models. Log-rank Chi squared testing was performed for survival analyses. All analyses were performed using Python version 3.11.5. The scripts are available at github.com/JustineCole (accessed on 27 August 2025).

## 3. Results

### 3.1. Summary Data

Table 1 and Supplemental Figure S1 summarize the demographic, lipid panel and outcome data in each dataset used in this study. All datasets had ~50% female with an average age of ~55 years. NHANES and ARIC had a higher percentage of diabetics (13% and 10%, respectively) and smokers (23% and 26%, respectively) than the UK Biobank (2% and 1%). In the ARIC dataset, 30% of individuals had ACVD, whereas only 10% in UK Biobank had ASCVD. Thus, it was decided to train and validate the models on the ARIC data, and to test on the UK Biobank data.

Despite the lower prevalence of ASCVD, mean and median systolic blood pressure, TC, TG and apoB were all substantially higher in UK Biobank than in the other datasets. HDL-C was also higher in UK Biobank. In UK Biobank and ARIC, NHDLC was higher than in NHANES.

### 3.2. Novel Phenotype System

Figure 2a and Table 2 show the algorithm by which individuals are assigned to one of the nine novel phenotypes and the lipid profile of each phenotype. Supplemental Figure S2 provides a visualization of the nine phenotypes in 3D space. In this figure, the nth group is concealed behind the other groups, and the NTH group was omitted so that the normolipidemic group is visible in the center. Table 3 and Figure 2b show the distributions of the nine phenotypes in the NHANES, ARIC and UK Biobank datasets, using NHANES and UK Biobank cutoffs for the UK Biobank cohort. In NHANES, the nth, normolipidemic and NTH phenotypes were the most prevalent at 24.6%, 17.9% and 17.7%, respectively. The least common were NtH and nTh at 2.4% and 4.4%, respectively. This is expected, because it is known that TG and HDLC are commonly inversely associated. However, the ntH and nTH had equal prevalences of 7.6% and the NTh phenotype was more prevalent than these at 8.9%. Given the higher median NHDLC in the ARIC dataset, it is not surprising that the NTH and NtH phenotypes were more prevalent in this cohort than in NHANES. Otherwise, the distribution in ARIC was similar to NHANES. The prevalences of each phenotype in the UK Biobank dataset were quite different when using the NHANES cutoffs, because of the aforementioned elevated lipid parameters in UK Biobank. Thus, the NTh and NTH phenotypes were the most prevalent at 24.4% and 20.8%, respectively, and the normolipidemic and nth phenotypes had a similar prevalence to the Nth group, at 14–15%. Meanwhile, when the UK Biobank cutoffs were used, the pattern of prevalence was more like the US population. This highlights the fact that reference values are not necessarily transferable and should ideally be determined for each population being studied.

Supplemental Figure S3a,b show how the nine phenotypes intersect with the prevalence of MetS, PCE scores and ASCVD-free survival. Overall, the UK Biobank dataset had lower prevalences of MetS, which is unexpected given the higher mean TG in this cohort. For analysis, we consider only the UK Biobanks phenotyping based on UK Biobank medians and IQRs (UKBu in the figure). The prevalence of MetS ranged from ~2% to ~80% in the different phenotypic groups across datasets. As would be expected, the highest prevalences of MetS were in the high TG and low HDLC groups (NTH and nTH), ranging from 57 to 80%. Groups with either high TG or low HDLC had 12–45% prevalence, but the groups with only elevated NHDLC or all low values (Nth and nth) had only 2–15% prevalence. Interestingly, the prevalence of MetS in the normolipidemic group was ~30% in the US cohorts and ~13% in UK Biobank. The PCE scores ranged from ~4% to 11%. As expected, the NTH group had the highest average PCE score, and the nth group had the lowest. Those groups with two indices elevated generally had higher risk scores than those with one or none elevated. Interestingly, in NHANES, the group with only low HDLC (ntH) had a borderline mean PCE score (7.5%). The NTH group had the steepest ASCVD survival curve, while the nth group had the shallowest. Of note, the nTh and Nth groups also had shallower survival curves than the normolipidemic group, with the ntH group overlying the normolipidemic group. Thus, singly elevated TG, NHLDC, or low HDLC alone appear not to significantly increase the risk of ASCVD events above normal. Only simultaneous abnormalities in two or more lipids appear to be associated with higher risk.

### 3.3. Predictive Models

The logistic regression models used to predict risk had the overall following form:

$$LI=\frac{1}{1+e^{-\left(B_{0}+B_{1}x_{1}+B_{2}x_{2}+B_{3}x_{3}\ldots +B_{m}x_{m}\right)}}$$

where *LI* is the lipid index, *x*_1_ to *x*_m_ are the features included in the model, *B*_1_ to *B*_m_ are the coefficients of the corresponding variables and *B*_0_ is the intercept. Table 4 provides the parameters for each model.

**Figure 3:**
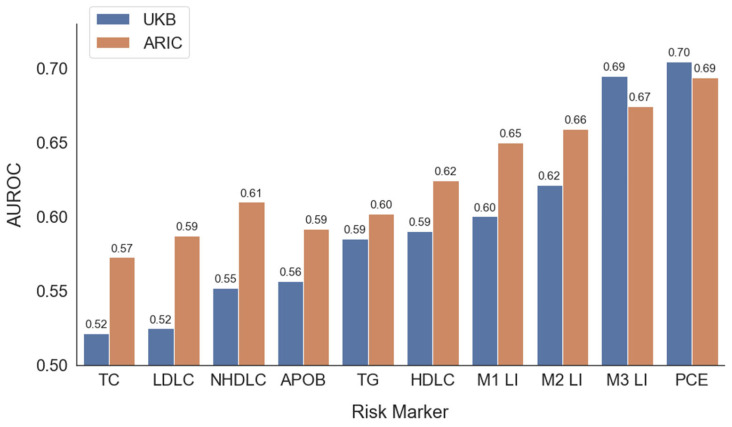
Area under the receiver operating characteristic curve for predicting atherosclerotic cardiovascular disease in UK Biobank (UKB) and ARIC databases.

Figure 3 displays the AUROC scores for commonly measured or calculable lipid markers, L1, L2, L3, and the PCE score for predicting ASCVD in the ARIC and UK Biobank datasets. The PCE had the highest AUROC scores with 0.70 in UK Biobank and 0.69 in ARIC. The AUROC scores of L3 were slightly lower, at 0.69 and 0.67, respectively. None of the other individual markers performed as well as a predictor of ASCVD than L1, which integrates all the different tests in the lipid panel. Inclusion of sex and age in the lipid indices and the PCE score afford these markers a great advantage in risk prediction due to the strong associations between male sex and older age with ASCVD risk. The other risk markers may interact with sex and age in unique ways and thus their true predictive power cannot be fully realized when examined alone.

Table 5 and Supplementary Table S1 provide the sensitivity and specificity, positive predictive value (PPV), negative predictive value and F1 score for predicting ASCVD of LDLC, apoB, TG, NHDLC, L3 and PCE in the UK Biobank and ARIC datasets using conventional cutoffs and the 80th percentile of the L3 score in NHANES (0.37) or UK Biobank (0.16), as well as optimized cutoffs. The conventional cutoffs provided very low sensitivity and much higher specificity for each biomarker. The optimized cutoffs were determined using univariate ROC curve analysis in the ARIC and UK Biobank training datasets, and cross-validation applied to a cost function that penalized false negatives to optimize sensitivity. This improved the F1 scores of all markers, but the PCE scores consistently performed the best, with L3 in a close second. The PPV and, consequently, F1 score are affected by the number of true positive values in the dataset. Only 10% of participants in UK Biobank had a CVD outcome, compared to 30% in ARIC. This explains why the PPV and F1 scores are higher for all markers applied to ARIC.

**Figure 4:**
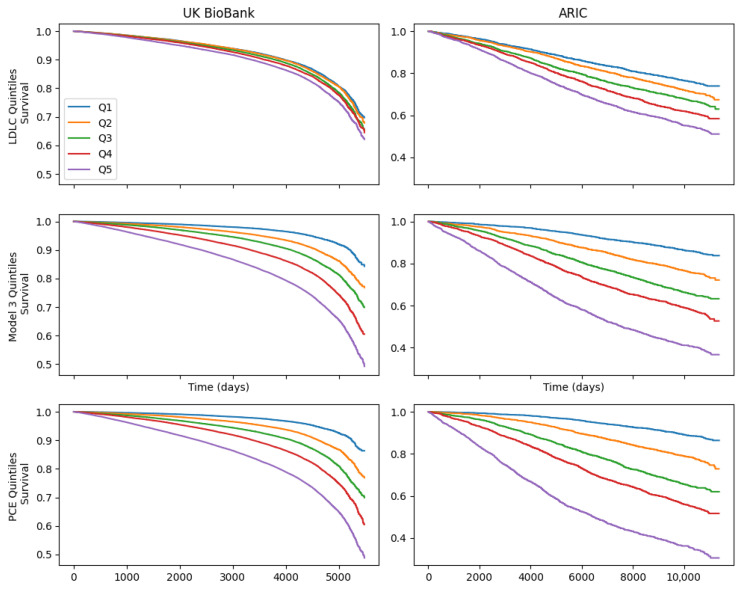
Comparison of atherosclerotic cardiovascular disease-free survival by low-density lipoprotein quintiles, model 3 score quintiles and pooled cohort equation score quintiles.

Figure 4 displays the survival curves for quintiles of LDLC, L3 and PCE in the UK Biobank and ARIC datasets, with ASCVD as the endpoint. Quintiles of the L3 and PCE scores provided much clearer separation, with log-rank Chi square scores for L3 of 9004 and 1634, and for PCE scores, 9796 and 2420 in UK Biobank and ARIC, respectively, versus 441.8 and 338.6 for LDLC quintiles.

The US guideline prescribes using a persistently elevated LDLC ≥ 160 mg/dL, a persistently elevated TG ≥ 175 mg/dL or an apoB ≥ 130 mg/dL as risk enhancer tests to inform the risk discussion in patients with LDLC of 70–190 mg/dL and 10-year risk scores of 5–20%. In those of this subgroup, an L3 score ≥37% in the ARIC dataset and ≥16% in UK Biobank correctly identified 53% and 44%, respectively, of patients who went on to develop ASCVD—more than any of the other risk enhancer tests at their prescribed cutoffs. Together, all the risk enhancers correctly identified >70% of positive cases in both UK Biobank and ARIC (Supplemental Figure S4a,b). The second part of Table 5 shows the performance metrics of these markers as risk enhancer tests at the prescribed cutoffs in the subgroup of patients with LDLC of 70–190 mg/dL and 10-year risk scores of 5–20%. It is clear from this analysis that these cutoffs are not optimized. For example, apoB ≥130 mg/dL has a relatively good specificity of ~90% but a sensitivity of only ~11% for identifying at-risk patients from among those at intermediate risk based on LDLC and PCE scores. Reducing the apoB cutoff to the optimized 87 mg/dL improved the F1 score to 0.50 in ARIC.

## 4. Discussion

In the present study, we sought to develop an approach for characterizing patterns of dyslipidemia or dyslipoproteinemia based on the non-linear relationships among multiple lipid markers and CVD risk. These non-linear relationships may be represented by (hyper)spherical coordinates in multidimensional space according to the number of markers included in the model. Given their routine availability in the clinical setting, we initially chose to use TG, NHDLC and HDLC, which make up the standard lipid panel, to illustrate the concept in three-dimensional space. Using median values of the markers from NHANES 1999–2018 [16], we described one normolipidemic and eight dyslipidemic patterns and showed how these patterns are differentially associated with MetS and ASCVD risk in NHANES, ARIC and UK Biobank [16,17,18]. We then went on to use the spherical coordinates, age and sex in a logistic regression model to provide a composite lipid index that may be used as a novel risk score or a risk-enhancing test during clinical assessment. The purpose of creating a phenotyping system that can be represented in three-dimensional space, and of using spherical coordinates rather than Cartesian coordinates, is that spherical coordinates allow one to imagine a sphere-like boundary that represents a composite decision limit.

One outcome of our study exemplifies why reference intervals and decision limits should be validated in the population in which they are to be used. We found that using the NHANES decision limits in the UK Biobank cohort produced an unlikely phenotype distribution. Secondly, we confirmed that while NHDLC elevation may occur in isolation, TG and HDLC derangements tend to occur simultaneously, and elevated TG is rarely found as an isolated derangement. However, in these three cohorts, 20–25% of individuals had either TG and HDLC above (-Th) or both below (-tH) the median, bearing in mind that the H index is 1/HDLC. Thus, elevated TG does not always go together with low HDLC, just as small, dense LDL is not always accompanied by elevated TG [21]. This highlights that fact that there are nuances involved in these commonly oversimplified relationships that are related to the lipid composition of lipoproteins. It further raises the point, which will be reiterated below, that what is true in summary for a group is not always true for an individual. Thus, clinical evaluations should ideally be individualized in every case, and algorithms, diagnoses and risk models used as guiding information rather than as decision points [5,22].

Approximately 30% of US individuals with normal lipid results had MetS, while those with isolated elevated NHDLC (Nth) had a lower prevalence of MetS than those that are considered normal. Given how our classification system works, the -th in this group may represent normal or low TG and normal or high HDLC, so it may be that this group had a lower mean TG and higher mean HDLC than the normolipidemic phenotype, in whom the TG and HDLC levels are within the IQR of each parameter. Similarly, the normolipidemic group had similar or higher mean PCE scores than the Nth, nTh and ntH groups. Both the Nth and the nTh groups had better ASCVD-free survival than the normolipidemic group, while the ntH group was similar to the normolipidemic group. This may be expected given the evidence that lipid-lowering below normal and below targets confers additional benefit [23]. Thus, the nth phenotype is expected to have the most favorable risk profile, and having only a single lipid abnormality is tolerable, whereas simultaneous abnormalities elevated ASCVD risk.

Fredrickson, Levy and Lees were the first to develop a dyslipoproteinemia phenotyping system based first on ultracentrifugal separation and then on electrophoresis [6]. This system proved invaluable to our fundamental understanding of lipoprotein metabolism [2,24,25,26,27,28] and to the link between genotype–phenotype relationships [29,30]. Due to the unavailability of the requisite lipoprotein separation methods in most clinical labs today, dyslipoproteinemia phenotyping is rarely performed in modern practice. De Graaf et al. developed an algorithm, based on apoB, that provides a FLL-like dyslipoproteinemia phenotype without the need for special laboratory techniques [7]. Gilliland et al. recently used this algorithm to investigate clinical associations in the UK Biobank dataset, and to explore novel genotype–phenotype associations [8]. Although the algorithm tended to be somewhat inaccurate in diagnosing FLL phenotypes, it proved valuable as a standalone phenotyping system, given the differential associations with clinical features and ASCVD risk, and the novel genotypes they were able to identify which contribute to polygenic disease. On the other hand, the reported high prevalence of remnant hypercholesterolemia, its lack of association with CVD risk, but strong associations with protein energy malnutrition and gout, suggest shortcomings of the algorithm that may reduce its value for patient management. Studies such as the one performed by Gilliland et al. are nevertheless important to highlight these limitations so that further refinement of phenotyping models can be achieved. In this study, we provide a proposal for how to refine such models. For example, by including retinol binding globulin along with apoB, TG and TC in a four-dimensional model, the algorithm of De Graaf et al. might be improved to be able to differentiate true remnant hypercholesterolemia from severe catabolic states [31]. Although we only used three dimensions in the current model described in this paper, the spherical coordinate system can be readily extended to many more dimensions.

Although dyslipoproteinemia phenotyping has proved valuable in research [7,8,32], Hegele suggests that clinical validation is required [33]. Berberich and Hegele propose that the FLL classification system is outdated as it was based on the notion that dyslipoproteinemias represent monogenic disorders, whereas it is now widely recognized that most abnormal lipoprotein phenotypes are due to polygenic influences, which interact with environment and lifestyle factors [34]. Hegele et al. suggested a simplified approach to dyslipidemia classification based on LDL-C and TG measurement [34]. We also previously described a system, using NHDLC and TG, which categorizes patients into the FLL-like dyslipoproteinemia categories [15]. Only dysbetalipoproteinemia (FLL Type III) could not be identified by this scheme, as this requires apoB measurement. Our current work moves away from FLL classifications and provides a novel phenotyping system, which includes HDLC. We did not include apoB in this example as it is not yet measured in routine clinical practice, but including apoB would convert the system from a dyslipidemia into a dyslipoproteinemia classification system, which would likely be beneficial, especially for risk stratification.

We also examined our novel spherical coordinates as a new integrated risk marker that is a composite of the three lipid tests in the standard lipid panel. Unlike previous models, we included HDLC, which appears to have an independent association with risk [35] and is used in several evidence-based risk ratios such as TG/HDLC and the Castelli Risk Indices: TC/HDLC and LDLC/HDLC [9,10]. Indeed, low HDLC was more common than high TG in the cohorts studied here and was associated with the shortest ASCVD-free survival in our study. Our system has the advantage of avoiding over-valuing the denominator—a limitation inherent in ratios. By including NHDLC, HDLC and TG in our model, we were able to produce an index (L1) with a higher AUROC for ASCVD in ARIC and UK Biobank than any single, routinely measured lipid test. By including age and sex, the third model resulted in an index (L3) that has comparable predictive power to the PCE score, which involves several clinical parameters and is not automatically calculated. In both the American and the European datasets, quintiles of L3 provided much greater separation of survival than quintiles of LDLC. We previously described a method to estimate the PCE score using data available to the clinical laboratory [14]. The current model does not attempt to approximate the PCE score but performs as well as the PCE score on a population level. Again, including apoB would likely be beneficial to the model given its superior association with ASCVD risk [36,37,38].

It is important to note that risk models developed using population data apply to predictions of outcomes for groups, and not precisely to individuals [39]. In the individual patient encounter, these scores should be used in the conversation around therapeutic decision-making, but individual characteristics and risk factors must also be considered. We noted that adding age to our model greatly improved the AUROC score. This is because atherosclerosis is a long-term process that starts early in life [22,40]. Thus, the risk associated with each marker should ideally be expressed as an interaction of that marker with age [38]. This finding reiterates the importance of engaging younger patients in conversations around healthy lifestyle choices to prevent ASCVD, even if their risk scores are low due to their young age.

We also examined the potential utility of our model for use as a risk-enhancing factor. In the current US guidelines, a persistently elevated LDL-C above 160 mg/dL, a TG above 175 mg/dL, an apoB above 130 mg/dL or a Lp(a) above 50 mg/dL are considered risk-enhancing factors that should guide the clinician–patient discussion to consider more seriously the need for lipid-lowering therapy or add-on therapy [3]. When applied in this context, we found that the L3 score was able to correctly identify significantly more individuals at risk than the other currently used risk-enhancing tests. It should be noted, however, that the prescribed cutoffs for the other risk-enhancing lipid tests do not appear to be optimal, so optimizing these and using them together may improve the quality of the information available to the clinician. The strengths of this work include that it uses data that is available for any patient with a standard lipid profile measurement and produces a risk score that performs similarly to the PCEs and a composite biomarker that outperforms the other lipid parameters as a risk-enhancing test. By equally weighting NHDLC, TG and HDLC, we avoided having any one value in the denominator of a ratio, which has mathematical advantages. Using a spherical coordinate system acknowledges the non-trivial physiological relationships among lipoproteins. In addition, it can be readily adapted to include more variables when making a single integrated risk score. Most importantly this new risk score could be automatically calculated by the laboratory information system, at no added cost, for all patients having a lipid panel test. Its use would not obviate the need to consider other risk factors like hypertension by the use of the PCE or other new risk scores like PREVENT, but it would automatically identify high risk patients based on lipids and thereby reduce the likelihood that these patients would not receive optimal care.

One limitation of our study is that the ARIC dataset is relatively small, so it was split 50:50 for training and validation of the models. This dataset was chosen as it has a more equal split of subjects with and without the target outcome compared with the UK Biobank dataset. A larger dataset would be ideal but was not available to us. Validation in the UK Biobank dataset has the limitations that this dataset is not representative of diverse ethnicities and there is a low prevalence of ASCVD. Another limitation is that different populations may require different thresholds for using the spherical coordinate system for assessing ASCVD risk, thus additional cohorts should be examined using this system to determine the generalizability of our findings or for optimizing thresholds for a particular population. A further issue is that our phenotyping system defined “normality” when all three parameters were within their IQRs. However, if at least one parameter was outside its IQR, the other parameters were labeled according to their relationship to the median. Thus, in the eight dyslipidemic phenotypes, we do not know which parameters were truly deranged and which were within their IQRs. A more precise classification system is possible but, in the interests of simplicity, was not attempted in this study. Finally, the use of AUROC scores only allowed us to show the possible utility of laboratory tests as binary classifiers. AUROC scores do not allow a full appreciation of the continuous and physiological relationships between these markers and ASCVD risk. The latter may be more important for monitoring disease progression and response to therapy and provides more individual level information, whereas the AUROC score provided the population level information we sought to illustrate here.

## 5. Conclusions

Our study proposes an alternative, holistic approach to dyslipoproteinemia phenotyping and ASCVD risk triage, moving beyond the oversimplified reliance on single lipid traits. Our key contribution is a novel risk-triage score based on a spherical coordinate system that integrates the full lipid panel with routinely available demographic data (age and sex). The main finding demonstrates that this model, which leverages the non-trivial mathematical interactions among markers, offers a more comprehensive assessment than current single-marker methods. This approach has direct clinical applicability for automated implementation within laboratory information systems, providing a new phenotype and risk factor score with interpretive comments to guide clinical decisions. This flexible methodology can also be expanded to multidimensional space. Limitations included the relatively small ARIC dataset, a lack of diverse ethnicities and low ASCVD prevalence in the UK Biobank validation cohort, and the use of AUROC scores, which provided population-level utility but did not fully explore the continuous physiological relationships critical for monitoring individual disease progression.

## Figures and Tables

**Table 1:** Summary demographic and clinical data for NHANES, UK Biobank and ARIC.

**NHANES**								
FEMALE		DIABETIC				SMOKER		
52%		13%				23%		
	COUNT	MEAN	STD	MIN	25%	50%	75%	MAX
**AGE**	10,942	54	8.8	40	46	53	61	70
**BMI**	10,790	29	6.8	14	25	28	33	130
**SBP**	10,359	126	18.7	64	113	123	136	225
**TC**	10,942	204	41.0	75	177	201	228	704
**HDLC**	10,942	54	16.8	6	42	51	63	226
**NHDLC**	10,942	150	41.6	23	121	146	174	685
**TG**	10,942	134	113.8	18	75	107	158	2742
**APOB**	5808	99	25.2	15	81	97	114	260
**ARIC**								
FEMALE		DIABETIC		SMOKER			ASCVD	
54%		10%		27%			30%	
**AGE**	14,195	54	5.8	44	49	54	59	66
**BMI**	14,182	28	5.4	14	24	27	30	66
**SBP**	14,188	121	19.0	61	108	119	131	246
**TC**	14,195	214	41.6	48	186	212	239	593
**HDLC**	14,195	52	17.0	10	40	49	61	162
**NHDLC**	14,195	163	43.8	10	133	160	190	521
**TG**	14,195	131	87.7	24	79	110	157	1926
**APOB**	14,190	93	28.8	12	73	90	110	294
**UK BIOBANK**								
FEMALE		DIABETIC		SMOKER			ASCVD	
57%		2%		1%			10%	
**AGE**	354,344	56	8.1	37	49	56	62	73
**BMI**	353,040	27	4.6	12	24	26	29	69
**SBP**	353,953	137	18.7	65	124	136	149	268
**TC**	354,344	228	41.2	58	199	225	253	597
**HDLC**	354,344	57	14.7	9	46	55	66	170
**NHDLC**	354,344	171	39.7	25	143	168	195	523
**TG**	354,344	151	88.8	20	90	128	185	998
**APOB**	352,608	106	23.1	40	90	105	121	200

BMI: body mass index; SBP: systolic blood pressure; TC: total cholesterol; HDLC: high-density lipoprotein cholesterol; NHDLC: non-HDLC; TG: triglycerides; APOB: apolipoprotein B; ASCVD: atherosclerotic cardiovascular disease.

**Table 2:** Lipid values associated with each phenotype.

Phenotype	NonHDL-C (mg/dL)	TG (mg/dL)	HDL-C (mg/dL)
normolipidemic	120–175	75–160	40–60
Proviso: The following do not meet all 3 criteria for normolipidemia			
nth	≤150	≤110	≥50
Nth	>150	≤110	≥50
NTh	>150	>110	≥50
NtH	>150	≤110	<50
nTh	≤150	>110	≥50
nTH	≤150	>110	<50
ntH	≤150	≤110	<50
NTH	>150	>110	<50

**Table 3:** Phenotype distribution in NHANES and UK Biobank.

Group	NHANES (%)	ARIC (%)	UK Biobank ^n^ (%)	UK Biobank ^u^ (%)
normolipidemic	17.9	15.2	14.8	16.6
nth	24.6	18.7	14.2	20.2
ntH	7.6	6.1	2.6	10.2
nTh	4.4	3.0	3.8	2.9
nTH	7.6	5.5	4.1	9.5
Nth	8.8	10.3	14.1	9.2
NtH	2.4	6.3	1.4	2.9
NTh	8.9	8.5	24.4	8.8
NTH	17.7	26.4	20.7	19.6

^n^: phenotype determined using NHANES cutoffs; ^u^: phenotype determined using UK Biobank cutoff.

**Table 4:** Model parameters.

LI	*x*_1_	*x*_2_	*x*_3_	*x*_4_	*B*_0_	*B*_1_	*B*_2_	*B*_3_	*B*_4_
L1	r	θ	φ	NA	−2.371	0.3550	−0.0107	−0.0268	NA
L2	L1	Female	Male	NA	−1.383	3.783	−0.9089	−0.3693	NA
L3	L1	Age	Female	Male	−4.756	3.718	0.0504	−0.2605	0.2615

LI: lipid index, L1: score using model 1; L2: score using model 2; L3: score using model 3; *x*_1_–*x*_m_: model features; *B*_1_–*B*_m_: coefficients of the corresponding features; *B*_0_: intercept; r: vector; θ: polar angle; φ: azimuthal angle.

**Table 5:** Performance of risk markers in ARIC and UK Biobank datasets using optimized cutoffs.

	CUTOFF	SENSITIVITY	SPECIFICITY	PPV	NPV	F1
	Risk Prediction					
	ARIC Dataset					
**APOB**	87 mg/dL	0.66	0.49	0.35	0.77	0.46
**LDLC**	131 mg/dL	0.66	0.47	0.35	0.76	0.46
**TG**	112 mg/dL	0.6	0.56	0.37	0.76	0.46
**NHDLC**	156 mg/dL	0.66	0.51	0.37	0.78	0.47
**L3**	26%	0.72	0.53	0.4	0.82	0.51
**PCE**	4.1%	0.76	0.51	0.4	0.83	0.52
	UK Biobank Dataset					
**APOB**	102 mg/dL	0.62	0.46	0.12	0.91	0.2
**LDLC**	133 mg/dL	0.63	0.4	0.11	0.9	0.19
**TG**	127 mg/dL	0.61	0.51	0.13	0.92	0.21
**NHDLC**	164 mg/dL	0.61	0.47	0.12	0.91	0.2
**L3**	11%	0.7	0.59	0.17	0.94	0.27
**PCE**	5.7%	0.69	0.61	0.17	0.94	0.28
	Risk Enhancer Tests					
	ARIC Dataset					
**APOB**	130 mg/dL	0.11	0.92	0.48	0.62	0.18
**LDLC**	160 mg/dL	0.3	0.75	0.44	0.63	0.36
**TG**	175 mg/dL	0.28	0.77	0.44	0.63	0.35
**L3**	37%	0.53	0.58	0.44	0.66	0.48
	UK Biobank Dataset					
**APOB**	130 mg/dL	0.12	0.89	0.16	0.85	0.14
**LDLC**	160 mg/dL	0.28	0.72	0.15	0.85	0.19
**TG**	175 mg/dL	0.38	0.64	0.16	0.86	0.23
**L3**	16%	0.44	0.65	0.18	0.87	0.26

LDLC: low-density lipoprotein cholesterol; APOB: apolipoprotein B; TG: triglycerides; NHDLC: non-high-density lipoprotein cholesterol; L3: score from model 3; PCE: pooled cohort equation.

## Data Availability

The NHANES and ARIC datasets analyzed during the current study are available in the NHANES and BioLINCC repositories, https://wwwn.cdc.gov/nchs/nhanes/, https://aric.cscc.unc.edu/aric9/, respectively (both accessed on 19 October 2025). The UK Biobank data that support the findings of this study are available from UK Biobank but restrictions apply to the availability of these data, which were used under license for the current study, and so are not publicly available. Data are, however, available from the authors upon reasonable request and with permission of UK Biobank.

## Supplementary Materials

The following supporting information can be downloaded at: https://www.mdpi.com/article/10.3390/jcm14217557/s1, Figure S1: Frequency distributions of age and lipid parameters in UK Biobank, NHANES and ARIC datasets.; Figure S2: Visualization of 9 phenotypes in 3D Space, Figure S3: Metabolic Syndrome, PCE Scores and ASCVD-free Survival in 9 Novel Phenotypes, Table S1: Performance of Risk Markers for Predicting ASCVD Using Conventional Cutoffs; Figure S4: Venn Diagrams of Performance of Risk Enhancer Tests.

## Abbreviations

The following abbreviations are used in this manuscript:

ASCVD	Atherosclerotic cardiovascular disease
apoB	Apolipoprotein B
CM	Chylomicrons
CVD	Cardiovascular disease
FLL	Fredrickson, Levy and Lees
HDLC	High-density lipoprotein cholesterol
LDL	Low-density lipoproteins
LDLC	LDL-cholesterol
Lp(a)	Lipoprotein (a)
PCEs	Pooled cohort equations
TC	Total cholesterol
TG	Triglyceride
VLDL	Very low-density lipoproteins
NHANES	National Health and Nutrition Examination Survey
ARIC	Atherosclerosis Risk in Communities
MetS	Metabolic syndrome
1/H	Inverse of HDL-C
LnTG	Natural logarithm of triglycerides
AUROC	Area under the receiver operating characteristic
PPV	Positive predictive value

## Appendix A

*Appendix A. ICD10 Codes Used to Define Atherosclerotic Cardiovascular Disease in the UK Biobank Dataset*

I200; I201; I208; I209; I210; I211; I212; I214; I219; I220; I221; I222; I228; I229; I230; I231; I232; I233; I234; I235; I236; I237; I238; I240; I241; I248; I249; I250; I251; I252; I253; I254; I255; I256; I257; I258; I259; I630; I631; I632; I633; I634; I635; I636; I638; I639; I640; I650; I651; I652; I658; I659; I660; I661; I662; I663; I668; I669; I700; I702; I708; I709.
