# Supplementary material for: Spherical Coordinate System for Dyslipoproteinemia Phenotyping and Risk Prediction
Source: J Clin Med. 2025 Oct 24;14(21):7557. doi: 10.3390/jcm14217557 (PMC12610519; doi:10.3390/jcm14217557)
Supplement: Supplementary file 1 [file jcm-14-07557-s001.zip › jcm-3870461-supplementary.pdf]

Supplemental Data

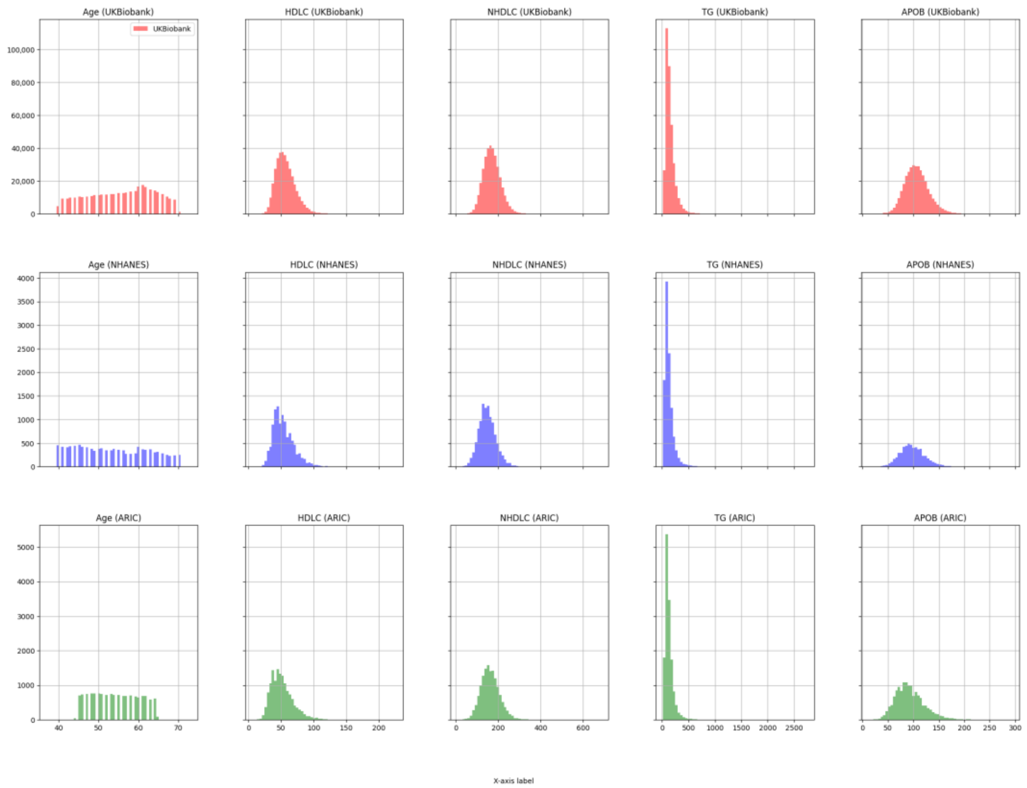

Figure S1. Frequency distributions of age and lipid parameters in UK Biobank, NHANES and ARIC datasets.

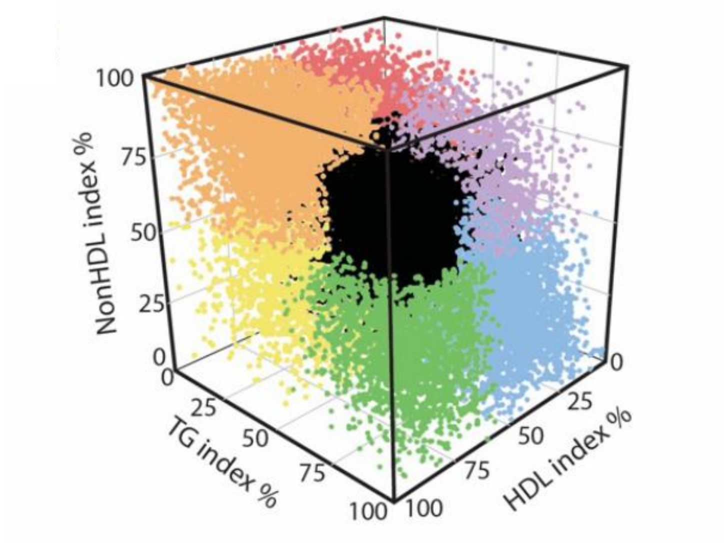

Figure S2. Distribution of NHANES data among the nine phenotypes and in three-dimensional space. The nth and NTH groups are not displayed. The nth group is hidden behind the others, and the NTH group was removed to display the normolipidemic group.

a

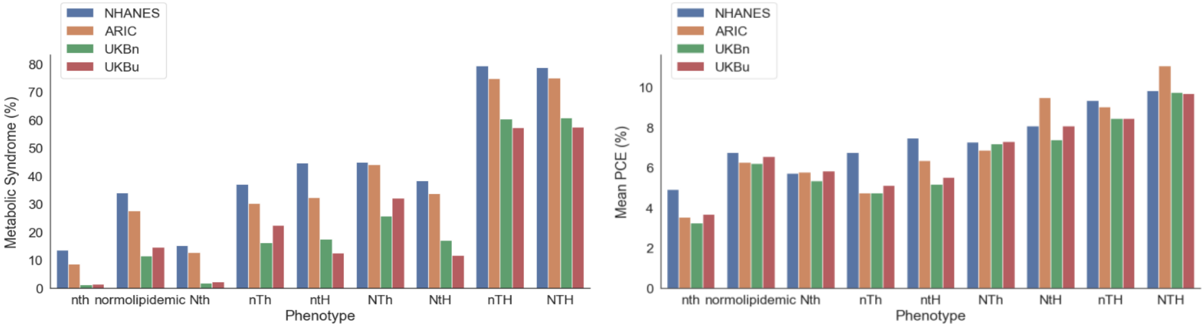

b

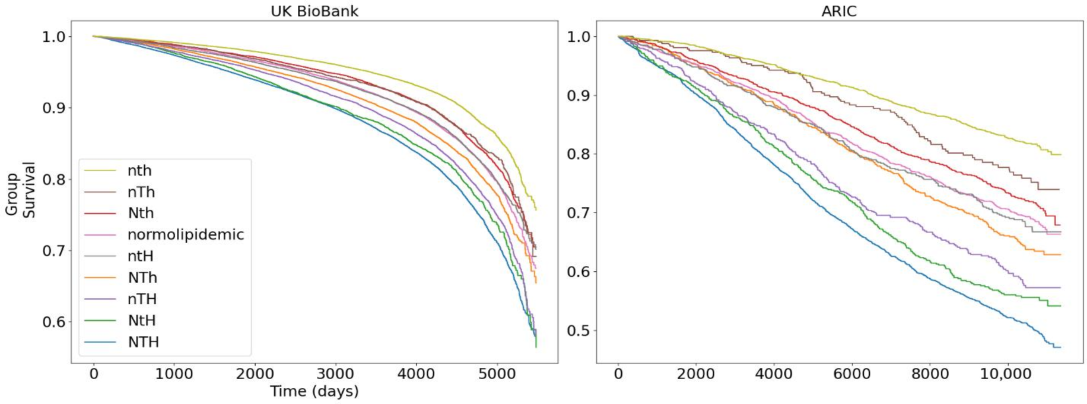

Figure S3. (**a**) Left: The frequency of the metabolic syndrome in each of the nine novel phenotype groups. Right: The mean pooled cohort equation score in each phenotype. (**b**) Survival without developing atherosclerotic cardiovascular disease in each phenotype in UK Biobank (left) and ARIC (right) databases.

Table S1. Performance of Risk Markers in ARIC and UK Biobank Datasets Using Conventional Cutoffs and the 80th Percentile of L3 in NHANES.

	CUTOFF	SENSITIVITY	SPECIFICITY	PPV	NPV	F1
			**Risk Prediction**			
			**ARIC Dataset**			
**APOB**	130 mg/dL	0.15	0.91	0.43	0.71	0.22
**LDLC**	160 mg/dL	0.37	0.76	0.39	0.74	0.38
**TG**	175 mg/dL	0.26	0.84	0.4	0.72	0.31
**NHDLC**	190 mg/dL	0.36	0.79	0.43	0.74	0.39
**L3**	37%	0.43	0.8	0.48	0.76	0.45
**PCE**	7.5%	0.54	0.73	0.46	0.79	0.49
			**UK Biobank Dataset**			
**APOB**	130 mg/dL	0.2	0.86	0.14	0.9	0.16
**LDLC**	160 mg/dL	0.33	0.71	0.12	0.9	0.17
**TG**	175 mg/dL	0.37	0.73	0.14	0.91	0.2
**NHDLC**	190 mg/dL	0.36	0.71	0.13	0.91	0.19
**L3**	16%	0.39	0.82	0.21	0.92	0.27
**PCE**	7.5%	0.59	0.7	0.19	0.94	0.28

LDLC: low-density lipoprotein cholesterol; APOB: apolipoprotein B; TG: triglycerides; NHDLC: non-high-density lipoprotein cholesterol; L3: score from model 3; PCE: pooled cohort equation.

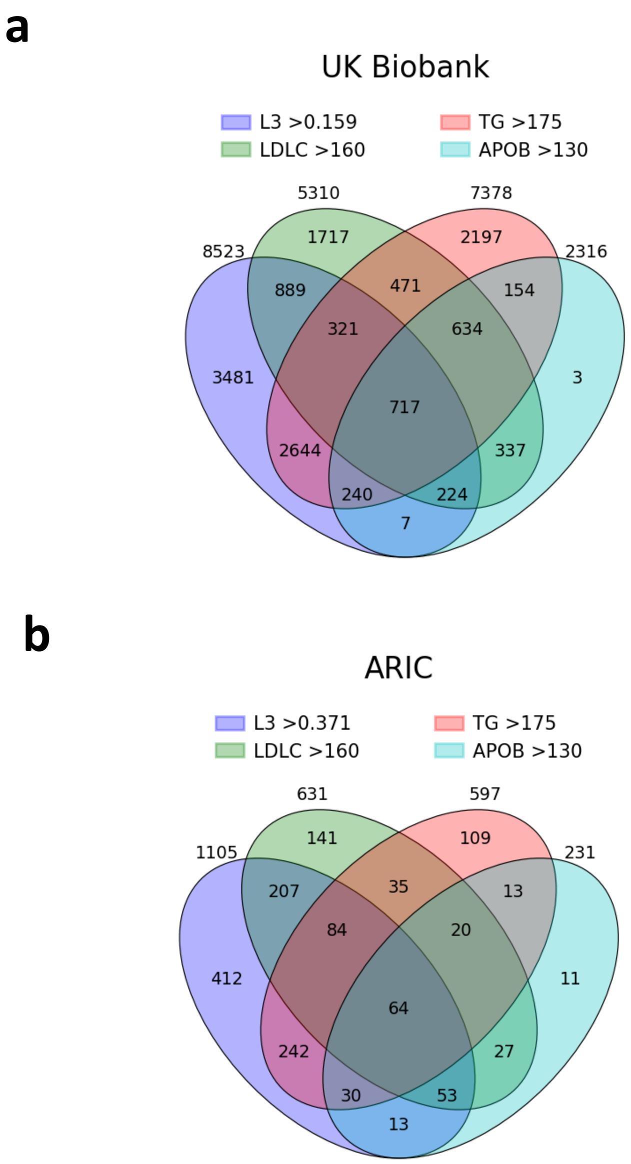

Figure S4. Venn diagrams comparing the total number of positive cases of atherosclerotic cardiovascular disease identified by four potential risk enhancer tests in (**a**) UK Biobank and (**b**) ARIC datasets.
